# Silk Fibroin Based Porous Materials

**DOI:** 10.3390/ma2042276

**Published:** 2009-12-09

**Authors:** Qiang Zhang, Shuqin Yan, Mingzhong Li

**Affiliations:** National Engineering Laboratory for Modern Silk, College of Textile and Clothing Engineering, Soochow University, No. 199 Ren’ai Road, Industrial Park, Suzhou 215123, China; E-Mails: zhangq12041008@163.com (Q.Z.); ysq_zq@163.com (S.Y.)

**Keywords:** *Bombyx mori* silk fibroin, porous materials, processing, application

## Abstract

Silk from the *Bombyx mori* silkworm is a protein-based fiber. *Bombyx mori* silk fibroin (SF) is one of the most important candidates for biomedical porous material based on its superior machinability, biocompatibility, biodegradation, bioresorbability, and so on. In this paper, we have reviewed the key features of SF. Moreover we have focused on the morphous, technical processing, and biocompatibility of SF porous materials, followed by the application research. Finally, we provide a perspective the potential and problems of SF porous materials.

## 1. Introduction

*Bombyx mori* silk is a naturally occurring polymer that has been used in textile production and as clinical suture for centuries [1]. As a kind of protein fiber, silk is usually produced within specialized glands after biosynthesis by epithelial cells, followed by secretion into the lumen of these glands where protein is stored prior to being spun into fibers [2]. Silk in its natural form is composed of a filament core protein, SF, and a glue-like coating consisting of a family of sericin protein. SF consists of heavy (H) and light (L) chain polypeptides of ~390 kDa and ~26 kDa, respectively, linked by a disulfide bond at the C-terminus of the two subunits, and associates with the H-L complex primarily by hydrophobic interactions [3,4,5]. The hydrophobic blocks tend to form *β*-sheets or crystals through hydrogen bonding and hydrophobic interactions, forming the basis for the tensile strength of SF [6,7]. These ordered hydrophobic blocks combine with the less ordered hydrophilic blocks to give rise to the elasticity and toughness of SF [8,9,10].

Porous three-dimensional materials, and the network structure materials, are composed of interconnected or closed pores. They possess some excellent characteristics, such as favorable mechanical properties and optic-electrical properties, good perm-selectivity, selective adsorption and chemical activity. Porous three-dimensional biomaterials provide a microenvironment for attachment, increase surface area, support a large cell mass, form an extracellular matrix and play an important role in manipulating cell functions in regenerative medicine [11,12,13,14]. Moreover, tissue engineering is an interdisciplinary and multi-disciplinary field that aims at the development of biological substitutes that restore, maintain, or improve tissue function [15,16,17,18]. In a typical tissue engineering approach, to control tissue formation in three dimensions (3D), a highly porous scaffold is critical. In addition to defining the 3D geometry for the tissue to be engineered, the scaffold provides the microenvironment (synthetic temporary extracellular matrix) for regenerative cells, supporting cell attachment, proliferation, differentiation, tissue neogenesis [16,17,19,20], formation of new extracellular matrix, and transportation of nutrients and metabolic wastes [21]. SF exhibits diverse structures, mechanical properties and biocompatibility. Based on these features, interest has arisen in the use of *Bombyx mori* SFs as starting materials for biomaterials and scaffolds for tissue engineering [2,11,22,23,24,25,26,27]. Therefore, three-dimensional SF porous scaffolds’ chemical composition, physical structure, and biologically functional moieties are all important for tissue engineering. Recently, there have been many reports about SF porous materials which have been widely investigated in controlled drug delivery system, anticoagulant blood materials, biosensors, artificial ligaments, artificial tendon and artificial skin, *etc.* [11,28,29,30]. In this paper, we will focus on SF based porous materials derived from *Bombyx mori* silkworms. Several preparation methods and different morphous of SF porous materials are reviewed as follows.

## 2. Silk Fibroin Processing

### 2.1. Solution of Silk Fibroin

Because SF is coated by sericin, degummimg is very important. High-purity SF fiber can be obtained easily from degummed silk [31]. SF can be dissolved with neutral solutions of salts such as LiSCN, LiBr, and CaCl_2_ [32,33]. Pure SF solution is prepared through dialysis for 3 days in deionized water.

In the processing of SF porous biomaterials, preparation of SF-based scaffolds with high porosity and interconnected homogeneous pores has become one of the major challenges. Several methods including salt leaching, freeze-drying, gas forming and freeze-drying/foaming have been developed to fabricate porous fibroin scaffolds [21,34,35,36]

### 2.2. Non-Woven Silk Fibroin Mats

Non-woven SF nets/mats/membranes can be prepared using SF with diameters in the range of several to tens of micrometers in their native or partially dissolved forms [37,38,39]. A process for producing non-woven SF nets/mats/membranes comprises the following steps: firstly, degumming and removal of the sericin; secondly, by a homogenization and drying step that yields three-dimensional, non-woven nets/mats/membranes [40]. Non-woven mats can also be obtained by electrospinning SF fibers with different diameters [41,42,43,44,45,46,47]. Electrospinning uses electrical forces to produce polymer nanofibers with diameters around fifty nanometers and arbitrary length. Electrospinning occurs when electrical force at the surface of a polymer solution or melt overcome surface tension and viscoelastic forces and create an electrically charged jet. When the jet dries or solidifies, an electrically charged fiber remains, which can be directed or accelerated by electrical force and then collected in sheets or other useful shapes [48]. The two methods as mentioned above are the predominant ways to obtain SF-based porous mats.Nnon-woven mats in particular, which have mass surface area and rougher topography for cell attachment, have been used in many fields.

### 2.3. Silk Fibroin Hydrogels

Hydrogels are three-dimensional polymer networks which are physically durable to swelling in aqueous solution but do not dissolve in these solution [49]. Hydrogels are formed from regenerated SF solution by a sol-gel transition in the presence of acid, ions, or other additives [50,51,52,53,54,55]. During the gelation process, SF experiences a structural transition from random coil to *β*-sheet due to enhanced hydrophobic interactions and hydrogen bond formation [52,53,54,56,57]. The processes for producing regenerated SF hydrogels are as follows: (a) silk is obtained from silk cocoons; (b) the sericin layer covering the silk fibers is removed; (c) the disulfide bonds are broken in order to obtain aqueous SF solutions; (d) the silk aqueous solutions are concentrated; (e) some acid, ions, or other additives are added; (f) after further processing, such as freeze-drying, microporous SF sponges are formed from hydrogels. Recently, many applications suggest the potential of porous hydrogels for cell culture and regenerative medicine [58,59,60,61,62]. Those will be reviewed in later sections.

### 2.4. Silk Fibroin Porous Sponges

Porous sponges are important tissue engineering materials. Regenerated SF solutions have been utilized in the preparation of porous sponges. SF porous sponges can be obtained using porogens, gas forming, and freeze-drying, freeze-drying/foaming, electrospun fibers [63]. Solvent-based sponges were prepared using salt (e.g., sodium chloride) or sugar as porogen. Porogens such as NaCL were added into SF aqueous solutions in disk-shaped containers, and then the containers were covered and left at room temperature for 24 hours, and then leaching the salt in water at room temperature for 24 hours and drying it [14]. The gas foaming method was completed by adding NH_4_HCO_3_ into SF aqueous solutions and sublimation of NH_4_HCO_3_ in hot water for 10 minutes [34]. Freeze-drying-based sponges were prepared using crosslinking agent, freezing and drying [35,31]. The freeze-drying/foaming method was a composite processing method to prepare 3-D SF scaffolds. Unlike the freeze-drying method, the ice/silk composites were firstly placed in the atmosphere at 20 °C for different times to make them partly thaw and then lyophilized leaving a porous material [36]. Electrospinning has also been one of the important methods to obtain SF porous scaffolds. Firstly, SF dope was prepared by dissolving lyophilized SF in 98% (v/v) formic acid for 4 h. Impurities and bubbles were removed by filtration and brief vacuum exposure. For electrospinning, the dope was put in a 10 mL syringe with a 22G stainless-steel syringe needle connected to a power supply. The dope flow rate was accurately controlled by a metering pump. A rolling metal drum was used as a collector for the sheet-like nanofibrous scaffolds and a metal bath filled with methanol was used as a collector for 3-D nanofibrous scaffolds. The electrospinning process was performed at room temperature and 60% humidity. Then NaCl particles were put into the SF nanofiber dispersion as porogens [63,19]. There are many ways to prepare the 3-D SF scaffolds. Those mentioned are is not all ways, but rather the predominating methods used to form the material.

## 3. Biocompatibility and Degradation of Silk Fibroin Porous Materials

As biomaterial, the “heterogeneity” or immunogenicity of a SF porous biomaterial is a crucial limitation for its clinical applications. Once a heterologous antigen enters the body, B cells [64,65,66], macrophages [67,27], dendritic cells [68,69,70,71] and mast cells [72,73,74,75,76] from our immune system are activated and produce antibodies and various cytokines targeting antigen epitopes on the biomaterials to attack and get rid of the “foreigners” by humoral and cellular immune responses. The biocompatibility of SF porous materials is an important first consideration.

Several primary cells and cell lines have been successfully grown on different SF porous materials to demonstrate a range of biological outcomes. SF porous materials are biocompatible when studied *in vitro* and *in vivo* [37,39,77,78]. Sofia *et al.* [79] investigated the effects of different freezing temperature regimes on SF protein 3D scaffold pore microstructure. Then the fabricated scaffolds were used as a 3D model to monitor cell proliferation and migration. Wang *et al.* [80] have examined a novel biomaterial consisting of a non-woven SF net for its ability to support the growth of human cells (endothelial, epithelial, fibroblast, glial, keratinocyte, osteoblast). The outcomes showed that the SF net is highly human cell-compatible and may be applicable for the vascularization of the newly formed tissue. Altman *et al.* [2] also examined the biocompatibility of three-dimensional (3D) nonwovens of SF in *β*-sheet form implanted into the subcutaneous tissue of C57BL6 mice, using sham-operated mice as controls. SF nonwoven nets may be excellent candidates for clinical applications since they both enjoy a long-lasting biocompatibility, inducing a quite mild foreign body response, but no fibrosis, and efficiently guide reticular connective tissue engineering. In recent years, some researchers have studied the immunogenity of SF biomaterials. The residual sericin plays a crucial role affecting the biocompatibility of SF porous materials. All biomaterials derived from non-autologous source will elicit some level of foreign body response (FBR) following implantation *in vivo* [78]. Small fibroin particles and soluble sericin protein extracted from native silk fibers did not induce significant macrophage activation [27]. While sericin did not activate macrophages by itself, it demonstrated a synergistic effect with bacterial lipopolysaccharide. Uff *et al.* [67] reported that sutures considerably inhibit macrophage behavior *in vitro*. Hollander *et al.* [72] showed that it is possible that natural medical silk surgical sutures could also induce hypersensitivity in some patients, such as asthma. Wen *et al.* [73,74] found that silk is the highly potent allergen that could activate mast cell degranulation and inflammation, but further biochemical analysis by Dewair *et al.* [75] and Zhaoming *et al.* [76] concluded that contamination from residual sericin (glue-like proteins) was the likely cause. The *in vitro* inflammatory response of degummed SF compared with polystyrene and poly(2-hydroxyethyl methacrylate) showed less adhesion of immunocompetent cells [81]. SF films [82] implanted *in vivo* and SF non-woven mats implanted subcutaneously in rats [78] induced a lower inflammatory response. Surface modification is also the important method to improve the performance of SF porous materials. For example, surface modiﬁcation with the integrin recognition sequence (Arg-Gly-Asp)RGDs can increase cell attachment [83]. Glucose-oxidase was immobilized on SF films for use as a glucose sensor [84], and so on.

Biodegradation behaviors of SF porous materials play an important role in regenerative biomedicine. Many *in vitro* and *in vivo* studies have showed that the degradability of SF porous biomaterials was related to the mode of processing and the corresponding content of *β-*sheet crystalline form [49]. A useful scaffold for tissue engineering materials should be biocompatible, as well as biodegradable [85,86]. Li *et al.* [87] investigated the degradation behavior of porous SF sheets by *in vitro* enzymatic experiments with α-chymotrypsin, collagenase IA, and protease XIV. With 1.0 U/mL protease XIV, 70% of the SF sheet was degraded within 15 days at 37 °C. When the fibroin sheet was exposed to collagenase IA, the amount of silk II crystalline structure in the sheets decreased slightly, and a small amount of silk I crystalline structure was formed. When protease XIV was used, almost all the Silk II disappeared, but the crystallinity increased overall because the amount of Silk I increased. During digestion with protease XIV, the pore size of the fibroin sheets increased with increasing degradation time, until the sheets finally collapsed and became totally shapeless. The average molecular weight of the products after degradation with the three enzymes followed the order protease XIV < collagenase IA < α-chymotrypsin. More than 50% of the products resulting from degradation with protease XIV were free amino acids. To investigate the degradation behavior of SF materials, Rebecca *et al.* [88] researched SF yarns. The yarns were incubated in 1 mg/mL protease XIV at 37 °C to create an *in vitro* model system of photolytic degradation. Results support that silk is a mechanically robust biomaterial with predictable long-term degradation characteristics by many detection methods. Wang *et al.* [89] implanted 3-D SF porous scaffolds into two kinds of rats and observed the morphous of the materials at different times. These results demonstrate that the *in vivo* behavior of the three-dimensional SF scaffolds is related to the morphological and structural features that resulted from different scaffold preparation processes. Gu *et al.* [90] have investigated the degradation behaviors of SF-nerve guidance conduits (SF-NGCs) *versus* SF fibers. The results collectively indicated that SF-NGCs were able to degrade at a significantly increasing rate, as compared to SF fibers, thus meeting the requirements of peripheral nerve regeneration.

In summary, SF porous material has proven biocompatibility and degradation *in vitro*. An important characteristic of SF is an increasing instability and solubility over time *in vitro* and *in vivo*, due to enzymolysis. Long-term stability and mechanical integrity are essential for cells that require sufficient time and stiffness to produce their tissue-specific matrix. Therefore, adjustment of degradation rate of the SF scaffolds material in order to match with the tissue regeneration is very necessary.

## 4. Application Studies of Silk Fibroin Based Porous Materials

*Bombyx mori* silk fibers have been used in the production of textile goods for centuries due to their characteristic luster, moisture absorbance and strength. Recently, SF porous material has been investigated in biomedical materials fields including bone and cartilage, skin tissue, vascular grafts, nerve repairing, ligaments and tendons. In addition to all the mentioned above, SF porous material was also studied in repairing of cornea, wound dressings, drug release, sensors and so on. All of these aspects exhibit the great prospects of SF porous materials for biomedical applications.

### 4.1. Bone and Cartilage

Bone tissue is a specialized form of connective tissue, which is composed of calcified extracellular matrix and bone cells including osteoprogenitor, osteoblasts, osteocytes and osteoclasts. The bone matrix consists of both an organic and inorganic matrix [91]. The biodegradability, distinguishing mechanical properties, and low inflammatory response of SF [30,80,92] ensure its role as one of the promising porous materials for osteogenic applications. Recently, SF porous material has been the primary biomaterial observed as bone and cartilage materials.

Fibers electrospun from aqueous solution of *Bombyx mori* SF, polyethylene oxide(PEO)and bone morphogenetic protein-2 (BMP-2) were prepared as a scaffold for human mesenchymal stem cells (hMSCs); *in vitro* culture in osteogenic media led to the formation of bone-like tissue. Addition of hydroxylapatite nanoparticles to the SF solution prior to electrospinning produced fibers with the nanoparticles embedded inside and was found to improve bone formation [30]. *In vivo* implantation of electrospun *Bombyx mori* SF fibers in calvarial defects in mice facilitated the complete healing of the defect with new bone within 12 weeks [93]. Similarly, *Bombyx mori* SF hydrogels have been used as scaffolds for bone tissue growth both *in vitro* and *in vivo* in rabbits without inflammatory effects [54,59,94]. SF non-woven mats were implanted in calvarial defects of rabbits for bone regeneration and resulted in complete healing with new bone at 12 weeks [42]. Aside from biologically generated bone as above, options to control hydroxyapatite mineralization on silk biomaterial matrices have also been reported [95]. The results suggest increased osteoconductive outcomes with an increase in initial content of apatite and BMP-2 in the SF porous scaffolds. The premineralization of these highly porous SF protein scaffolds provided enhanced outcomes for the bone tissue engineering [96]. SF was also blended with poly (L-aspartate) as a template for the growth of apatite crystals [95]. Calcium-phosphate (Ca-P) coatings, which comprise a range of minerals found naturally in bone, have been shown to reduce the fibrous encapsulation layer, enhance direct bone contact and stimulate differentiation of bone marrow stromal cells along the osteogenic lineage [97,98,99].

Survival rate of seeded cells is very critical for bone tissue engineering, but the seeded cells inside the scaffold may not survive sufficiently to repair a large critical sized defect. These cells likely recruited host cells to participate in new bone formation and defect repair. There are some possible mechanisms for this recruitment. First, the seeded cells secreted extracellular matrix, followed by capillary sprouting and vascular invasion into the newly synthesized matrix which brought in additional host cells to promote the healing process [100,101]. Another likely explanation is that the seeded cells secreted growth factors which could recruit invading reparative cells from the surrounding host tissue engaged in the new bone formation more rapidly and the interaction between cells and the mineral on the scaffold was responsible for this [102,103]. Moreover, highly porous scaffolds which performed the role of a temporary matrix for anchorage dependent cells are an important factor in the success of tissue engineering. Vascularization which is also critical for osteogenesis, seeded cells, as well as resident host cells is essential to the repair success of critical sized defects.

### 4.2. Skin Tissue

Skin tissue is the biggest organ in human’s body. It plays a crucial role in protecting the human body against the environment, dehydration, and infectious agents. Several studies show that SF porous material can accelerate wound healing, improve adhesion and spreading of normal human keratinocytes and fibroblasts, upgrade the growth and development of skin tissue. Proteins are among the most successful materials applied as skin grafts [104]. Fibrin is a kind of good skin substitutes [105,106].

Min and colleagues carried out a series of studies to investigate the potential of SF electrospun matrices for accelerating the early stages of wound healing [45,46,107]. The adhesion and spreading of some cell and cell lines were evaluated. For keratinocytes, but not fibroblasts, coating with collagen I promoted cell adhesion and spreading. Laminin coating stimulated cell spreading, but not cell attachment. Cell adhesion and spreading on fibronectin-coated silk matrices was found comparable to that on BSA-coated matrices [44]. In wound healing studies, SF nanofibers, microfibers, and films [45], nanofibrous matrices promoted human oral keratinocyte adhesion and spreading. The authors attributed the results to the higher surface porosity and surface area-to-volume ratio of nanofibers, which formed a higher surface area for cell attachment. Fibroblast spreading was significantly improved when silk matrices were treated with water vapor rather than methanol [107].

In another study, chitin was blended with SF to fabricate composite fibrous scaffolds for skin tissue engineering, due to the wound healing effects of chitin [81]. The chitin/SF blends at varied ratios were electrospun into nanofibrous matrices and evaluated for initial cell attachment and spreading [44]. Yoo *et al.* [108] fabricated chitin/silk hybrid matrices at various blend ratios using side-by-side electrospinning and compared these to chitin/silk blend matrices for keratinocyte adhesion and spreading. Chitin/silk (75%/25%) blend matrices demonstrated higher support for adhesion and spreading than all chitin/silk hybrid matrices, thus demonstrating the strong potential of blend matrices as skin regeneration substitutes. Yeo *et al.* [109] investigated the potential of collagen/silk blend porous matrices for skin tissue engineering. Surprisingly, pure collagen and pure SF matrices exhibited better results in terms of human keratinocyte attachment and spreading than collagen/silk blend or hybrid matrices irrespective of the mixing ratios. Increased adhesion of keratinocytes was observed on chitin/silk blend matrices compared to pure chitin matrices, although there was no difference for fibroblasts [110]. In our studies, SF porous materials were prepared by freeze-drying. The L929 cells can attachment and proliferation well in the SF porous materials, and it was similar to that of in collagen materials. No signs of cellular lysis, intracellular granulation or cell morphological changes were observed. In conclusion, SF porous materials have great potential for skin tissue repairing. But reduction of the scar and regeneration of coil gland still are face-problem to solve in skin repairing.

### 4.3. Vascular Grafts

In these years, attention to tissue engineered vascular grafts has increased due to the inclusion of vascular cells. SF porous matrices have potential as vascular graft matrices due to their ability to support the attachment, proliferation and differentiation of vascular cells and resist shear stress and pressure from simulated blood flow. Fibers electrospun from aqueous solutions of *Bombyx mori* SF and PEO were used as scaffolds for human aortic endothelial cells (HAECs) and human coronary artery smooth muscle cells (HCASMCs); in both cases *in vitro* culture in endothelial growth medium led to the formation of vascular tissues within a week [43].

Many attempts have been made to develop small-diameter blood vessels due to increasing demands for vessel transplants, but these approaches have almost all failed. Greatly reduced graft patency was observed when cell-free synthetic prostheses were utilized for small diameter arteries, such as coronary and infragenicular vessels [111,112,113,114]. Bondar *et al.* [115] have investigated endothelial cell (EC) responses to nano- and micro-scale silk fibers in terms of cell morphology, proliferation, formation of intercellular contact, and expression of adhesion molecules. Outcomes revealed no significant differences between micro- and nanofibrous scaffolds. Also, interactions between ECs and SF matrices were investigated through the expression of specific transmembrane receptor molecules. The results of real-time PCR revealed significant up-regulation of integrin-β_1_ in ECs grown on nanofibrous compared to microfibrous scaffolds. In addition, the formation of new focal adhesions (FAs) and polarization at the leading edge indicated a stronger migratory state of ECs on nanofibrous rather than microfibrous matrices. The authors attributed this to increased integrin expression, which may activate signal transduction to increase FAs, cell attachment, polarization and hence migration.

Soffer *et al.* [116] have developed SF into porous tubular structures. The tube was tested and compared to those from a previous study of SF porous mats by Ayutsede *et al.* [47]. The average burst strength of the tubular scaffolds (811 mmHg) was greater than those prepared with collagen (71 mmHg) [117]. However, further development is needed to reach the gold standard of the saphenous vein whose burst strength is 1,800 mmHg [118]. Following Soffer’s work, the evaluation of the biological potential of these electrospun SF porous matrices for vascular grafts was determined [119]. The proliferation, metabolic viability, morphology and phenotype of human aortic endothelial cells (HAECs) and human coronary artery smooth muscle cells (HCASMCs) on 2-D electrospun SF porous matrices were examined. The results showed that electrospun SF porous matrices have good biocompatibility. Thus, the need for further research into both the biological and mechanical properties was demonstrated. Future work with SF small-diameter vascular grafts will need to focus on co-cultures of endothelial and smooth muscle cells in a tubular, perfusion environment to more closely mimic the *in vivo* environment.

### 4.4. Nerve Grafts

Peripheral nerve repair represents a common clinical challenge, and the current gold standard for treating large nerve defects involves the implantation of nerve auto-grafts that is limited by graft availability, secondary deformities, and potential differences in tissue structure and size [120,121,122,123,124,125,126,127]. It has previously reported on good *in vitro* biocompatibility of SF fibers with peripheral nerve tissues and cells [128,129]. Several authors have reported that nerve conduits (NC) releasing neurotrophic factors can enhance nerve regeneration across long nerve gaps [130,131,132,133]. Nerve growth factor (NGF)-loaded nerve conduits-SF (SF-NC) were prepared from aqueous solutions of NGF and SF (20%, w/w), which were air-dried or freeze-dried (freezing at −20 or −196 °C) in suitable molds. Control experiments with differently dried NGF-lactose solutions did not evidence marked protein aggregation, loss of ELISA-reactivity or PC12 cell bioactivity [128]. In order to explore the feasibility of using purified SF to construct artificial nerve grafts, it is necessary to evaluate the biocompatibility of SF material with peripheral nerve tissues and cells. Results indicate that SF has good biocompatibility with dorsal root ganglia and is also beneficial to the survival of Schwann cells without exerting any significant cytotoxic effects on their phenotype or functions [129]. Then, they developed a novel biomimetic design of the SF-based nerve graft (SF graft) which was composed of a SF-nerve guidance conduit (NGC) inserted with oriented SF filaments. The SF graft was used for bridge implantation across a 10-mm long sciatic nerve defect in rats, and the outcome of peripheral nerve repair at six months post-implantation was evaluated by a combination of electrophysiological assessment, FluoroGold retrograde tracing and histological investigation. The examined functional and morphological parameters show that SF grafts could promote peripheral nerve regeneration with effects approaching those elicited by nerve autografts which are generally considered as the gold standard for treating large peripheral nerve defects [134]. However, involving the implantation of nerve auto-grafts, research gold standard for treating large nerve defects is still endeavored. For examples, the methods of preparation never material, some new starting material obtained for never material, the interaction between material and organism and so on.

### 4.5. Ligaments and Tendons

There is a need to develop ligament and tendon scaffolds that simultaneously possesses optimal strength, a porous structure and a biocompatible microenvironment. Therefore, competent scaffolding materials are needed, and these ideally should fulfill the following requirements [135]: biodegradability, biocompatibility, superior mechanical properties and maintenance, biofunctionality, and processability.

Engineered tissues can serve as high-fidelity models for controlled studies of cell function and tissue development under normal and pathological conditions [136]. Due to their excellent strength, biocompatibility, and biodegradability, SF fibers may be an exciting candidate material for ligament and tendon scaffolds. Recently, research on *Bombyx mori* silk on tissue engineering ligament exhibits a better solution. Bone marrow-derived mesenchymal stem cells (BMSCs) and anterior cruciate ligament fibroblasts (ACLFs) on combined SF porous scaffolds for ligament tissue engineering application were studied to compare the cellular responses. The results indicated that BMSCs were found to be a better cell source than ACLFs for the further study of ACL tissue engineering whatever the cellular response *in vitro* and *in vivo* [137]. Some people have also wanted to develop a new practical ligament scaffold by synergistic incorporation of the silk, a knitted structure, and a collagen matrix. The efficacy for ligament tissue engineering was investigated *in vitro* and in animal models. The outcomes demonstrated that the knitted silk + collagen sponge scaffold improves structural and functional ligament repair by regulating ligament matrix gene expression and collagen fibril assembly, and the concept of an ‘‘internal-space-preservation’’ scaffold is proposed for the tissue repair under physical loading [138]. Consequently, *Bombyx mori* silk porous material is a potential candidate scaffold material for tissue-engineered ligaments when used alone or combined with other materials [11,138,139,140]. However, as tissue-engineered ligaments, *Bombyx mori* SF materials still have to face great challenge. For instance, strength of SF porous materials was not enough to meet the ligaments and tendons. Long-term stability and mechanical integrity of SF porous materials are not controlled for cells which require sufficient time and stiffness to produce their tissue-specific matrix.

### 4.6. Drug Delivery

Drug delivery is the method or process of administering a pharmaceutical compound to achieve a therapeutic effect in humans or animals. Drug delivery technologies were used in modifying drug release profile, absorption, distribution and elimination for the benefit of improving product efficacy and safety, as well as patient convenience and compliance [141]. However, many medications such as peptides and proteins, vaccines, and gene based drugs, in general, may not be delivered using conventional routes. *Bombyx mori* SF materials for drug delivery may improve the drug delivery situation. *Bombyx mori* SF is a protein soluble in water, and, when processed into scaffolds, results in a biomaterial with excellent mechanical properties, slow bio-degradation and well established biocompatibility [78]. So, SF has been suggested as a platform for drug delivery either in the form of films [142,29] or as genetically engineered silk-elastine hydrogels [143,144] and other SF or SF blended hydrogels [55,145,146]. Some kinds of SF Porous 3D scaffolds were also prepared for drug delivery. To evaluate the feasibility of controlled insulin-like growth factor I (IGF-I) releasing SF scaffolds in the context of cartilage repair. IGF-I loaded porous SF scaffolds have the potential to provide chondrogenic stimuli to hMSC [147]. SF porous scaffolds with interconnective pores, carrying embedded microparticles that were loaded with insulin-like growth factor I (IGF-I), were prepared by a porogen leaching protocol. The potential of SF scaffolds for drug delivery were demonstrated by embedment of the poly (lactide-co-glycolide) microparticles into the scaffolds [148]. Several researchers used SF 3D scaffolds as a platform to control drug release and they found that SF scaffolds were exciting candidates for controlled release [149,150,151].

Because SF protein might be susceptible to enzymatic degradation or cannot be absorbed into the systemic circulation efficiently due to molecular size and charge issues to be therapeutically effective. For this reason, we still have many problems in further research. Current efforts in the area of drug delivery include the development of targeted delivery in which the drug is only active in the target area of the body (for example, in cancerous tissues) and sustained release formulations in which the drug is released over a period of time in a controlled manner from a formulation. Types of sustained release formulations include liposomes, drug loaded biodegradable microspheres and drug polymer conjugates.

### 4.7. Other Application Studies

Besides all those mentioned above, SF porous materials were also studied in some other fields. For example, glucose oxidase was immobilized onto the blended membrane of poly (vinyl alcohol) (PVA) and regenerated silk fibroin. The morphology and application to glucose biosensor were investigated [152]. Moreover, SF porous materials were regarded as cornea material [153] and tracheal scaffolds [154] and so on. In summary, application research of SF porous material demonstrated its great potential and role in biomaterial field.

## 5. Further Prospects

With development of SF porous material, more and more novel SF porous materials will be fabricated. But, there are still some problems should be solved:

(1) SF can be processed into diverse morphologies to meet the different needs. Novel methods should be originated to create a wide variety of exciting SF porous materials.
(2) Biodegradation rate should be controlled and made it match with the growth of organism.
(3) Surface modification also should be used to improve biocompatibility of SF porous materials. To develop SF porous biomaterials for clinical usage, the materials with lower immunogenicity or without the immunogenicity should take into consideration.
(4) Make sure that more and more new SF promising porous materials could be applied in clinical medicine.
